# Developing prognostic models for cholesterol-related genes linked with immune infiltration in prostate cancer

**DOI:** 10.3389/fgene.2025.1604113

**Published:** 2025-09-26

**Authors:** Jie Wu

**Affiliations:** Key Laboratory of Hydrodynamics (Ministry of Education), School of Ocean and Civil Engineering, Shanghai Jiao Tong University, Shanghai, China

**Keywords:** prostate cancer, immune-related genes, cholesterol, immunity, prognosis

## Abstract

**Background:**

Prostate cancer has a high incidence and a low 5-year survival rate. We aimed to combine cholesterol- and immune-related genes to screen prostate cancer prognosis-related genes and construct a prognostic risk model.

**Methods:**

We obtained publicly released clinical data of prostate cancer through The Cancer Genome Atlas. Cholesterol- and immune-related genes were separately collected from the mSigDB and ImmPort databases. The prognostic model based on the immune-cholesterol-related differentially expressed mRNAs (DEmRNAs) network was constructed by univariate and multivariate Cox regression methods. Gene set enrichment analysis (GSEA), mutation landscape analysis, and immune infiltration analysis were carried out to investigate the role of immune-cholesterol-related DEmRNAs in prostate cancer.

**Results:**

We identified 11 immune-cholesterogenic-related DEmRNAs (C2orf88, TRPM4, SAPCD2, RHPN1, RAC3, APOF, PTGS2, TSPAN1, KLK4, ENTPD5, and C1orf64) as risk factors that were related to the occurrence and development of prostate cancer by bioinformatics analysis. Immune infiltration analysis suggested immune-cholesterol-related DEmRNAs may act in an immunomodulatory role for treatment decisions. The proportion of plasma cells, memory resting CD4 T cells, and neutrophils in the low-risk group was significantly higher than that in the high-risk group (p < 0.05). The GSEA revealed DEmRNAs were enriched in 58 Kyoto Encyclopedia of Genes and Genomes (KEGG) pathways, consisting of hematopoietic cell lineage, hypertrophic cardiomyopathy, and the JAK-STAT signaling pathway. The Gleason score of the high-risk group showed a significant difference from that of the low-risk group after clinical data analysis (P < 0.05).

**Conclusion:**

The prognostic risk model and nomogram constructed based on the immune-cholesterol-related genes had a great prognostic performance for prostate cancer.

## Highlight

• Cholesterogenic-related genes can divide prostate cancer patients into two subgroups with different prognoses.
• Cholesterogenic- and immune-related genes were enriched in smooth muscle contraction, cGMP-PKG pathway, and focal adhesion.
• Eleven genes (C2orf88, TRPM4, SAPCD2, RHPN1, RAC3, APOF, PTGS2, TSPAN1, KLK4, ENTPD5, and C1orf64) were used to construct a risk model.
• The risk model had good prognostic performance for patients at 1 year, 2 years, and 3 years.
• The risk score was an independent prognostic factor of prostate cancer.

## Introduction

Prostate cancer is the cancer with the highest morbidity and mortality rate among men in China. There are projected to be 2.3 million new cases globally by 2024 (Culp et al., 2020). Prostate cancer growth and development are controlled by 5α-dihydrotropis, which plays its biological role by binding to the androgen receptor. When 5α-dihydrotrophil binds to hormone ligands, the receptor is detached from the helper protein and transferred, then binds to the androgen response element located in the promoter region of genes involved in cell proliferation, and escapes from apoptosis (Dehm and Tindall, 2006). Studies have confirmed that androgens bind to the androgen receptor, activating androgen receptor signaling and promoting the development of prostate cancer (Hou et al., 2021b). At present, androgen deprivation therapy is the preferred treatment for prostate cancer. The treatment slows disease progression by lowering circulating androgens to castration levels. However, androgen deprivation therapy eventually leads to the development of drug resistance, and the disease process cannot be stopped (Powers et al., 2020). The treatment of advanced prostate cancer will change dramatically with the future development of genomics and bioinformatics. Informational biomarkers are urgently needed.

Cholesterol is not only a precursor of male hormones; it is also a key component of the lipid raft, which is the main platform for cancer signaling regulation (Ding et al., 2019). Some cancers, including prostate cancer, have elevated cholesterol levels, and higher cholesterol levels make raft domains less fluid. Changes in the structure of raft domains may promote tumor growth by stimulating related signaling pathways (Hryniewicz-Jankowska et al., 2019). Elevated cholesterol promotes tumor growth and reduces apoptosis of prostate cancer cells through the AKT signaling pathway (Singh et al., 2017). Feeding prostate cancer mice a high-cholesterol diet led to increased tumor growth (Jamnagerwalla et al., 2018). However, the molecular mechanism of cholesterogenic-related genes in prostate cancer and whether they can be used as prognostic molecular markers of prostate cancer need further study.

The successful use of immune checkpoint inhibitors in a variety of cancers, including bladder cancer, has aroused interest in tumor immunity (Gibney et al., 2016). The impact of immune components and immune cell types on prostate cancer is complex (Zhao et al., 2019). The immune cells play a key role in the progression and metastasis of prostate cancer (Messex and Liou, 2023). An elevation in CD4^+^ T cell numbers has been observed in prostate tissue from prostate cancer patients relative to control samples (Hu et al., 2015). Macrophages are positively correlated with postoperative aggressive pathological features of prostate cancer (Strasner and Karin, 2015). Higher numbers of CD8^+^ cytotoxic T lymphocytes were found in CCR6-deficient mice that had bone metastasis from prostate cancer (Chinetti-Gbaguidi et al., 2017). Immune cell infiltration patterns in the prostate tumor microenvironment, including Tregs and M1/M2 macrophages, have been identified as adverse prognostic indicators for prostate cancer (Andersen et al., 2021). Of note, clinical studies have confirmed that manipulating cholesterol can reshape the immune landscape and play a positive role in tumor treatment (Huang et al., 2020). Cholesterol metabolism has been shown to impact the function of CD8^+^ T lymphocytes (Yang et al., 2016). Additionally, cholesterol homeostasis is vital for macrophage function (Guo et al., 2018). These findings reveal the important role of immune cells as well as their relationship with cholesterol metabolism in prostate cancer. However, the cross mechanisms related to both cholesterol metabolism and immune cells in prostate cancer progression have not been fully clarified.

In this study, we aimed to use the large amount of sequencing and clinical data in The Cancer Genome Analysis (TCGA) database, combined with cholesterogenic-related genes and immune-related genes, to screen for prostate cancer prognosis-related genes. These genes were used to construct a prognostic risk model to provide some new insights into the mechanism research and prognosis prediction of prostate cancer (Supplementary Figure 1).

## Materials and methods

### Data collection

RNAseq (Log2 (FPKM +1)) and clinical data from the TCGA database (Rosenbloom et al., 2015) for GDC TCGA Prostate Cancer (PRAD) were downloaded. A total of 551 samples (52 paracancer samples and 499 tumor samples) with matched sequencing and clinical data were obtained. Combined with the Gencode database (Harrow et al., 2012), the Ensembl_ID was converted to a Symbol_ID to obtain the mRNA expression value.

The gene expression dataset GSE70769 was acquired from the Gene Expression Omnibus (GEO, https://www.ncbi.nlm.nih.gov/geo/). It included 94 tumor tissue samples and was sequenced on the GPL10558 platform. This dataset was utilized for external validation of the prognostic model.

Genes belonging to the molecular signatures database gene sets “REACTOME_CHOLESTEROL_BIOSYNTHESIS” (n = 24) in the mSigDB database (Karasinska et al., 2020) were used as cholesterogenic genes. The immune gene set was obtained from the ImmPort database (Bhattacharya et al., 2018) and matched with the TCGA data set to create a 1,315 immune gene expression matrix.

### Consensus clustering analysis of cholesterogenic genes

The ConsensusclusterPlus algorithm (version 1.50.0) (Wilkerson and Hayes, 2010) was used to cluster all tumor samples based on the cholesterogenic gene matrix. The parameters were set as maxK = 6, pItem = 0.8, clusterAlg = “hc”, distance = “spearman”. The cumulative distribution function (CDF) was used to identify the most reasonable number of clusters, and the corresponding sample set was used to obtain the subtype of cholesterogenic genes. The log-rank statistical test was performed to analyze the relationship between cholesterogenic genes subtypes and prognosis, and Kaplan–Meier (K–M) survival curves were constructed.

### Screening differentially expressed mRNAs

The T-test provided by the limma package (version 3.10.3) (Smyth, 2005) of R software was used to test the mean expression difference between tumor and paracancer samples. The adjusted P-value <0.05 and | logFC (fold change) | > 1 were set to screen differentially expressed mRNAs (DEmRNAs). ggplot2 was used to visualize the results.

The Pearson correlation coefficient (PCC) between DEmRNAs and cholesterogenic genes was calculated to identify the cholesterogenic-related DEmRNAs using the cor() function in R. We also calculated the PCC between DEmRNAs and immune genes to obtain the immune-related DEmRNAs. A P-value <0.01 and |r| > 1 were set as the threshold. Finally, cholesterogenic-related DEmRNAs and immune-related DEmRNAs were intersected to obtain immune-cholesterogenic-related DEmRNAs.

### Enrichment analysis of immune-related DEmRNAs and cholesterogenic-related DEmRNAs

ClusterProfiler (Yu et al., 2012) was used to perform the Gene Ontology (GO) (Kanehisa and Goto, 2000) and Kyoto Encyclopedia of Genes and Genomes (KEGG) (Carbon et al., 2019) enrichment analysis of immune-related DEmRNAs and cholesterogenic-related DEmRNAs. Enrichment terms with count ≥2 and P-value <0.05 were identified as significant terms.

### Construction and verification of a prognostic risk model

After removing TCGA tumor samples with a survival time of less than 30 days, 496 samples remained. We randomly divided the sample into a training set and a validation set in a ratio of 3:2. Univariate Cox regression analysis using the survival package (version 4.0–2) was used to identify immune-cholesterogenic-related DEmRNAs that were significantly correlated with progression-free interval (PFI). The best λ values were obtained by using the LASSO Cox regression model (Tibshirani, 1997) of the R package glmnet (version 4.0–2) (Engebretsen and Bohlin, 2019). The ten-fold cross-validation was conducted, and the lambda.min was used for selecting the optimal combination of characteristic genes for model construction.

The prognostic risk model was constructed by combining the gene expression value in each tumor sample, the PFI, and the PFI time of each sample according to the following formula: Risk score = βgene1 × exprgene1 + βgene2 × exprgene2 +…+ βgenen × exprgenen. In the formula, β represents the prognostic correlation coefficient of each gene in LASSO regression, while expr represents the expression value of the corresponding gene. The samples with a risk score ≥ optimal cut-off point were classified as the High_risk group. The Low_risk group was defined as samples with a risk score < optimal cutpoint.

To verify the accuracy of the risk model, the risk model with the internal validation set and the entire data set, respectively, were reconstructed based on the internal validation and entire data sets, as well as the external validation dataset, and the β value obtained from the training set was used. A K–M curve was used to visualize whether there was a significant difference in PFI between the two groups. SurvivalROC was used to collate the survival time and survival status of the samples. The receiver operating characteristic curves (ROC) (version 1.0.3) (Heagerty et al., 2000) of 1-year, 2-year, and 3-year patient survivals were described. We also calculated the corresponding area under the curve (AUC). Then, the samples were divided into high and low expression groups according to the optimal expression level of the samples, and a log-rank statistical test was performed. Finally, a K–M survival curve was drawn to visualize the relationship between model genes and patient prognosis.

### Independent analysis of the prognostic model and establishment of a nomogram

We performed univariate and multivariate Cox analyses to screen independent prognostic factors. The enrolled factors included Gleason score, pathologic N, pathologic T, age, therapy outcome, radiation therapy, and risk score. A log-rank test was used for difference analysis, and a P-value <0.05 was selected as the threshold. The nomogram was plotted using the rms nomogram function (version 6.1-0) (Zhang et al., 2017) in R, combined with independent prognostic factors. The predictive power of the nomogram was evaluated using calibration curves.

### Statistical analysis of clinical data

To compare the differences in clinical characteristics (age, pathologic N, pathologic T, and Gleason score) between the High_risk and Low_risk groups, GGStatsplot (version: 0.5.0) was used to calculate the proportion of each clinical characteristic. The chi-square test was used to compare the difference between the two groups. A P-value <0.05 was considered the threshold value.

### Screening of differentially expressed genes between the High_risk and Low_risk groups

For differentially expressed gene (DEG) screening, the T-test in the limma package (version 3.10.3) (Smyth, 2005) in R was used. An adjusted P-value <0.05 and |logFC| > 0.585 were selected as the threshold.

Gene set enrichment analysis (GSEA) was used to compare the different pathways between the High_risk and Low_risk groups using clusterProfiler (version 3.16.0) (Yu et al., 2012) in the R package. c2. cp.kegg.v7.5.1.symbols.gmt in the MSigDB database was set as the enrichment background. GSEA was performed after sorting according to the logFC of all genes obtained by grouping comparison. An adjusted P-value <0.05 was considered the threshold.

### Immune infiltration analysis

The Cibersort algorithm (Newman et al., 2015) was applied to analyze the infiltration of 22 kinds of immune cells in groups using tumor tissue expression profile data. The gene expression characteristic template was the LM22 dataset provided by the Cibersort website. perm = 100 and QN = F were set as the parameters.

### Mutation analysis

The somatic mutation file called by TCGA’s mutect software was downloaded. Maftools (version 2.0.16) (Mayakonda et al., 2018) of the R package was used to draw a summary diagram of the groups, and statistical analysis of somatic mutation information was performed.

## Results

### Consistent clustering analysis

**FIGURE 1:**
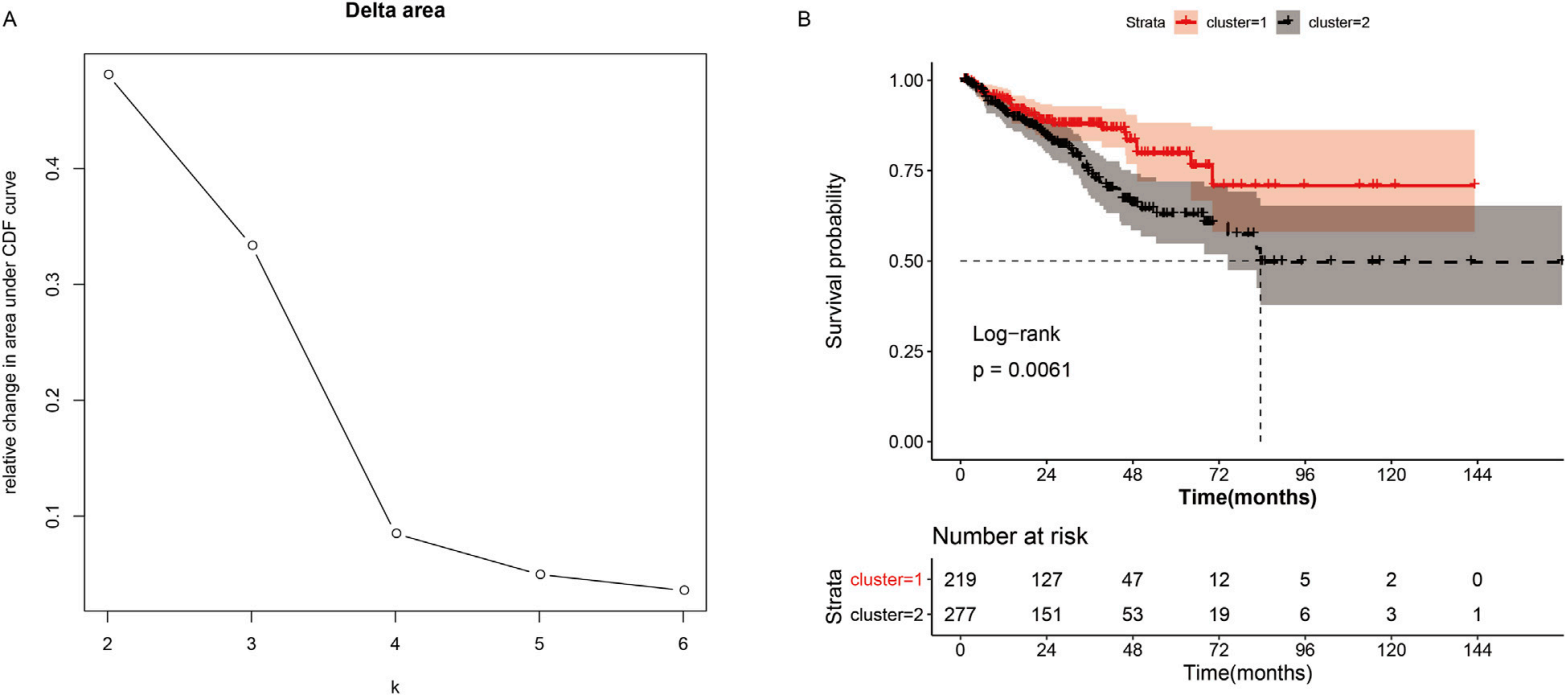
Consistent cluster analysis of cholesterogenic genes. **(A)** The cumulative distribution function curves when the cluster number changes from k to k+1. The optimal k = 2. **(B)** Survival curves of patients in cluster-1 and cluster-2.

Consistent clustering analysis of cholesterogenic-related genes revealed that tumor samples should be divided into two subtypes (Figure 1A). The K–M curve revealed that the survival probability (P = 0.0061) showed a significant difference (Figure 1B) between the two clusters. This result prompted us to continue to investigate the relationship between cholesterol levels and prognosis in prostate cancer patients.

### Screening of cholesterogenic-related DEmRNAs and immune-related DEmRNAs

**FIGURE 2:**
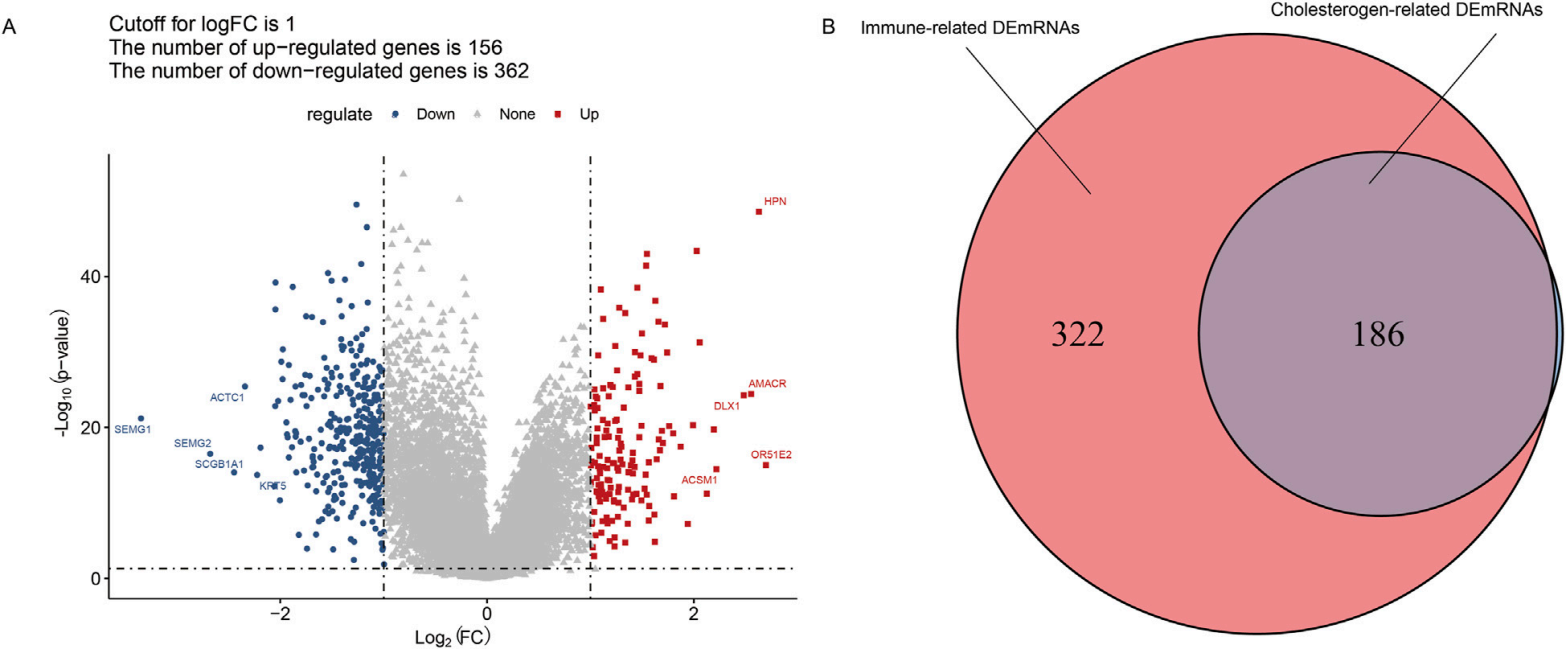
Screening of immune-cholesterogenic-related differentially expressed mRNAs (DEmRNAs). **(A)** The volcano map of DEmRNAs between the tumor and the adjacent tissues. **(B)** Venn analysis of immune-cholesterogenic-related DEmRNAs.

The results of differential analysis showed that there were 518 DEmRNAs (156 upregulated and 362 downregulated) between the tumor and the adjacent tissue (Figure 2A). Moreover, to screen the cholesterogenic-related DEmRNAs and immune-related DEmRNAs, we calculated the PCC between DEmRNAs, cholesterogenic-related genes, and immune-related genes. There were 508 immune-related DEmRNAs and 187 cholesterogenic-related DEmRNAs. To further screen the immune-cholesterogenic-related DEmRNAs, the cholesterogenic-related DEmRNAs and immune-related DEmRNAs were intersected. Finally, 186 immune-cholesterogenic-related DEmRNAs were obtained (Figure 2B).

### Functions of cholesterogenic-related DEmRNAs and immune-related DEmRNAs

**FIGURE 3:**
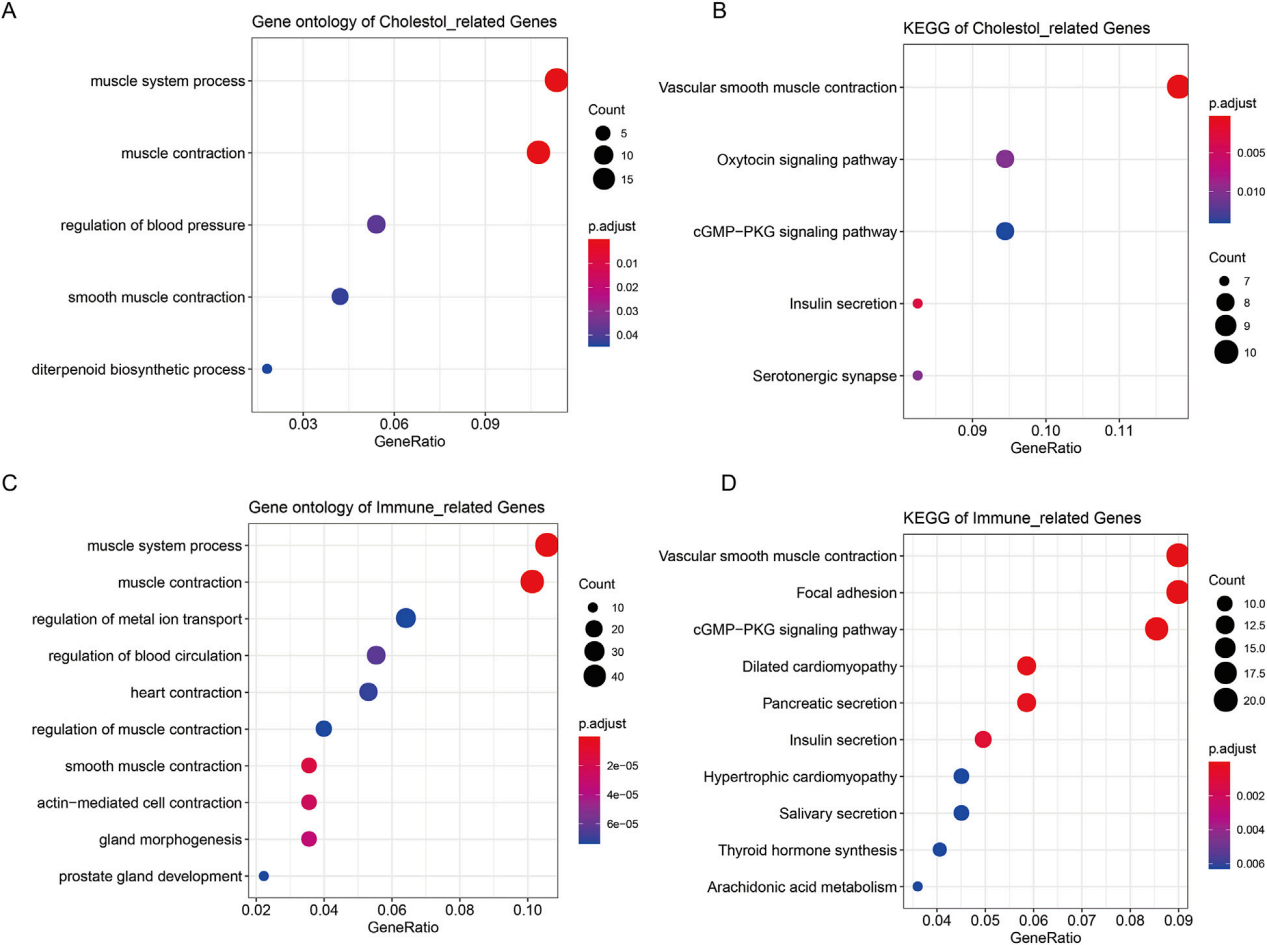
Enrichment analysis of cholesterogenic-related DEmRNAs and immune-related DEmRNAs. **(A)** The GO analysis terms of cholesterogenic-related DEmRNAs. **(B)** The KEGG analysis of cholesterogenic-related DEmRNAs. **(C)** The GO analysis of immune-related DEmRNAs. **(D)** The KEGG analysis of immune-related DEmRNAs.

The 187 cholesterogenic-related DEmRNAs were enriched in five GO terms, including muscle system process, muscle contraction, regulation of blood pressure, smooth muscle contraction, and diterpenoid biosynthetic process (Figure 3A). The cholesterogenic-related DEmRNAs also participated in five KEGG pathways, including the cGMP-PKG signaling pathway, vascular smooth muscle contraction, oxytocin signaling pathway, insulin secretion, and serotonergic synapse (Figure 3B).

The 508 immune-related DEmRNAs were enriched in 304 GO terms, including muscle system process, muscle contraction, and regulation of metal ion transport (Figure 3C). These mRNAs were also enriched in 21 KEGG pathways, such as the cGMP-PKG signaling pathway, vascular smooth muscle contraction, and focal adhesion (Figure 3D).

### The risk model

**FIGURE 4:**
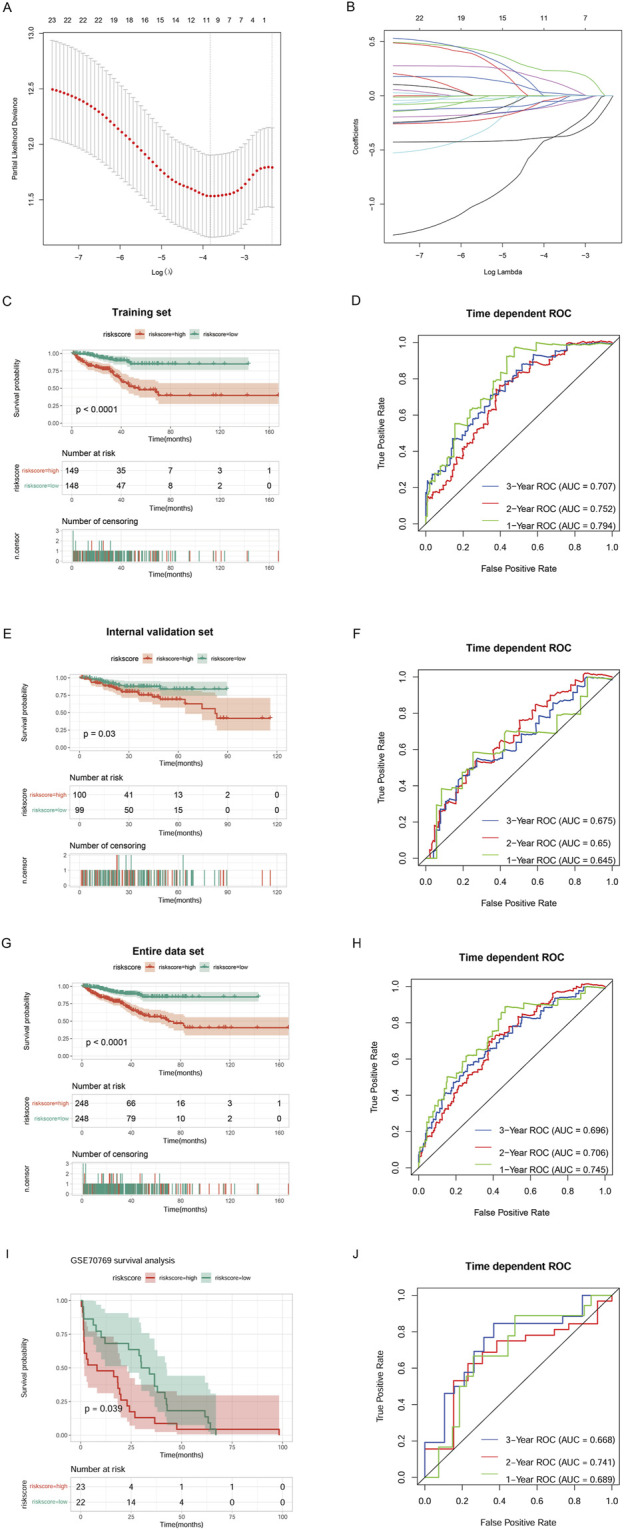
Construction of a risk model. **(A)** Selection of the tuning parameter (lambda) in the LASSO model by 10-fold cross-validation based on the minimum criteria for OS. **(B)** LASSO coefficient profiles. **(C)** The progression-free interval (PFI) of the TCGA training set. **(D)** Survival-dependent ROC curve validation at 1 year, 2 years, and 3 years of prognostic value of the prognostic index in the TCGA training set. **(E)** The PFI of the TCGA validation set. **(F)** Survival-dependent ROC curve validation at 1 year, 2 years, and 3 years of prognostic value of the prognostic index in the TCGA validation set. **(G)** The PFI of the entire TCGA data set. **(H)** Survival-dependent ROC curve validation at 1 year, 2 years, and 3 years of prognostic value of the prognostic index in the entire TCGA data set. **(I)** The PFI of the external validation dataset GSE70769. **(J)** Survival-dependent ROC curve validation at 1 year, 2 years, and 3 years of prognostic value of the prognostic index in the external validation dataset GSE70769.

Immune-cholesterogenic-related DEmRNAs were used to screen the PFI-related genes. The result indicated that 25 genes were significantly correlated with PFI. Further LASSO Cox regression analysis revealed that only 11 PFI-related genes (C2orf88, TRPM4, SAPCD2, RHPN1, RAC3, APOF, PTGS2, TSPAN1, KLK4, ENTPD5, and C1orf64) were selected as the optimized prognostic markers (Figures 4A,B).

Then, we combined the 11 PFI-related genes with the training set to construct a risk model. K–M survival analysis revealed that the High_risk group’s prognosis was significantly worse than the Low_risk group’s (P < 0.0001). ROC curves showed that our model had good prognostic performance for patients at 1 year (AUC = 0.794), 2 years (AUC = 0.752), and 3 years (AUC = 0.707) (Figures 4C,D). We verified the performance of the prognostic risk model using the internal validation set (Figures 4E,F), the entire data set (Figures 4G,H), and the external validation dataset GSE70769 (Figures 4I,J), and consistent results were obtained. These data indicated that the constructed risk model had high performance for predicting prognosis.

### The independent prognostic factors

**FIGURE 5:**
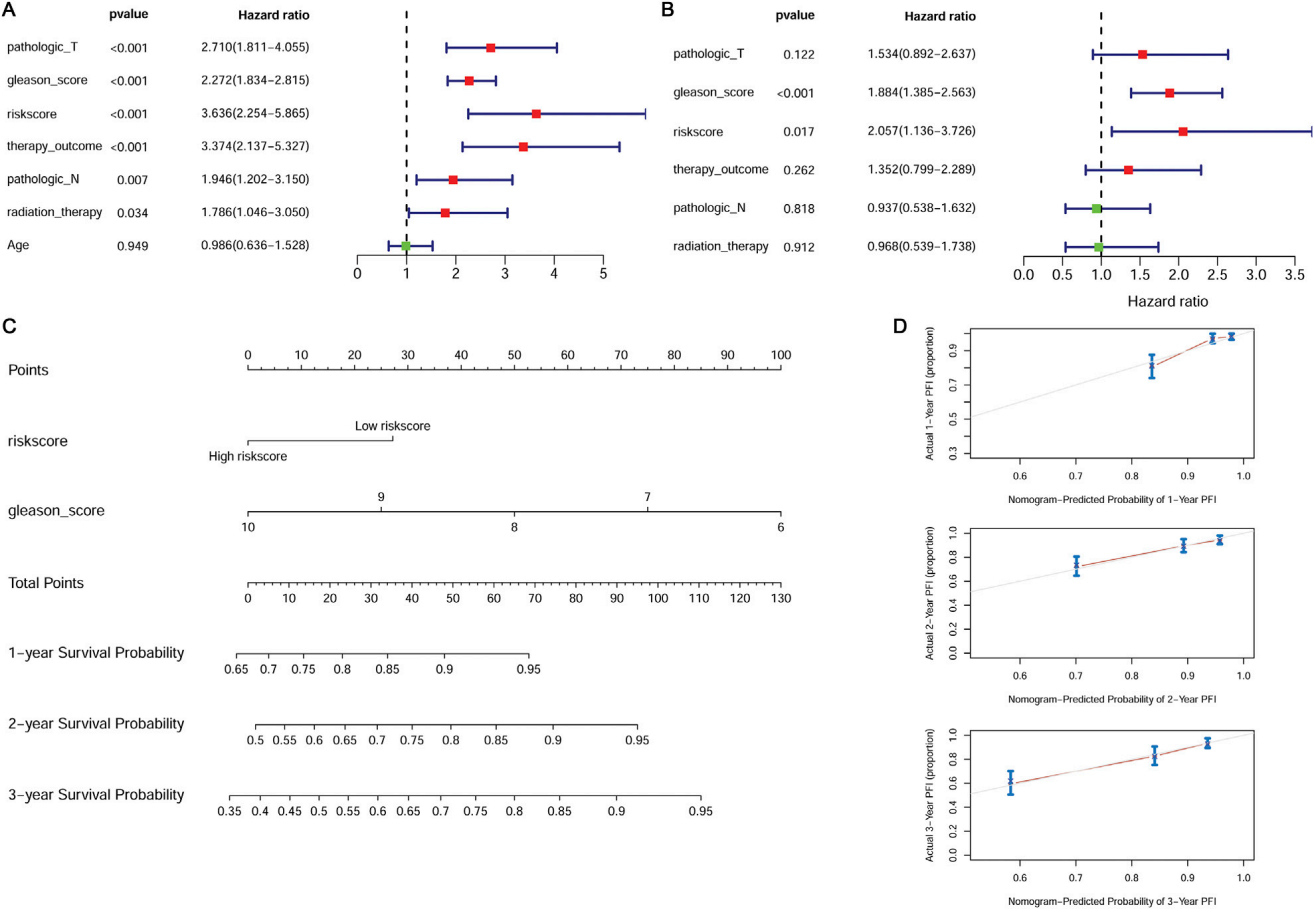
Independent prognostic validation of the risk model genes. **(A)** Forest plot of univariate Cox regression analysis. **(B)** Forest plot of multivariate Cox regression analysis. **(C)** The nomogram combined with the Gleason score and the risk score. **(D)** The calibration plot for internal validation of the nomogram.

Univariate and multivariate Cox regression analyses were performed to screen the independent prognostic clinical factors. Univariate Cox regression analysis revealed that risk score, pathologic T, pathologic N, Gleason score, therapy outcome, and radiation therapy were significantly related to prognosis (P < 0.05) (Figure 5A). Multivariate Cox regression analysis showed the Gleason score and risk score were significantly correlated with prognosis (P < 0.05) (Figure 5B) and were considered independent prognostic factors.

We constructed a nomogram combined with the Gleason score and the risk score (Figure 5C). The calibration curve was used to assess the predictive power of the nomogram. The prognosis predicted by the nomogram showed a good agreement with the actual prognosis (Figure 5D).

### Comparison of clinical data

The proportions of clinical factors were compared between the High_risk and Low_risk groups. The proportion of T3 patients was highest in the High_risk group, while the proportion of T2 patients was highest in the Low_risk group (P < 0.05) (Supplementary Figure 2A). There were significantly more patients older than 65 years in the High_risk than in the Low_risk group (P < 0.05) (Supplementary Figure 2B). The Gleason scores of the two patient groups also showed a significant difference (P < 0.05). The High_risk group had the most patients with a Gleason score of 9, while the Low_risk group had the most patients with a Gleason score of 7 (Supplementary Figure 2C). The number of patients at stage N1 was significantly higher in the High_risk group than in the Low_risk group (P < 0.05) (Supplementary Figure 2D).

### GSEA between the groups

**FIGURE 6:**
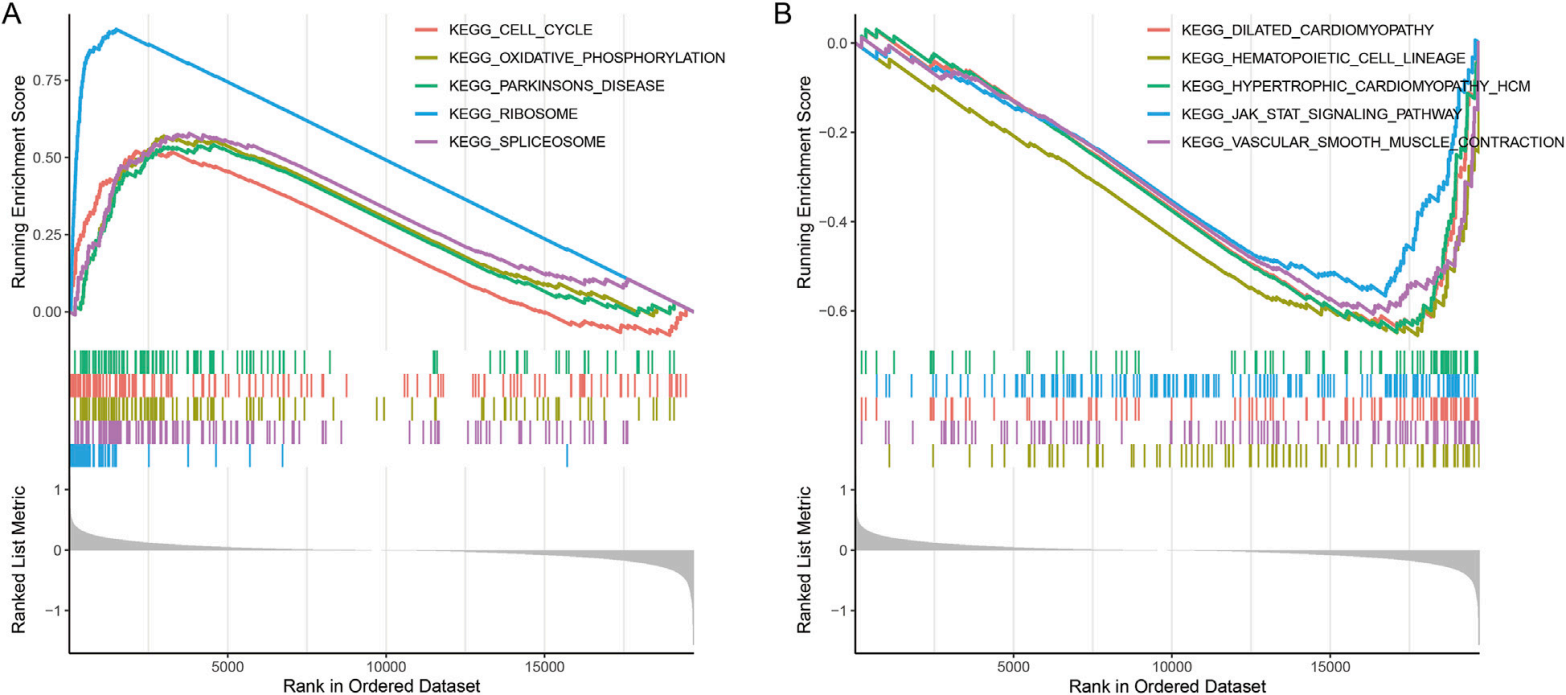
Enrichment plots of differentially expressed gene sets from gene set enrichment analysis (GSEA). **(A)** The top five KEGG pathways that were negatively related to the High_risk group. **(B)** The top five KEGG pathways that were positively related to the High_risk group.

There were 167 DEGs (27 upregulated and 140 downregulated) screened. GSEA revealed that DEGs were enriched in 58 KEGG pathways. Fourteen pathways that were negatively related to the High_risk group included cell_cycle, oxidative_phosphorylation, parkinsons_disease, ribosome, spliceosome, and so on (Figure 6A). Forty-four pathways that positively related to the High_risk group included vascular_smooth_muscle_contraction, dilated_cardiomyopathy, hematopoietic_cell_lineage, hypertrophic_cardiomyopathy, jak_stat_signaling_pathway, and so on (Figure 6B).

### Immune infiltration analysis

The plasma cells, memory resting CD4 T cells, and neutrophils showed a significantly higher fraction in the Low_risk group than in the High_risk group (P < 0.05). The regulatory (Tregs) T cells and M2 macrophages showed a significantly higher fraction in the High_risk group than in the Low_risk group (P < 0.05) (Supplementary Figure 3).

### Mutation of two groups

The top five genes with mutation frequencies in the High_risk group included TP53, SPOP, TTN, FOXA1, and MUC16 (Supplementary Figure 4A). The top five genes with mutation frequencies in Low_risk patients included SPOP, TTN, TP53, KMT2D, and KDM6A (Supplementary Figure 4B).

## Discussion

Cholesterol metabolism has been shown to play an immunomodulatory role in tumors. Targeted therapies targeting cholesterol metabolites in cancer cells and immune cells are considered a promising approach (Huang et al., 2020). We discussed the feasibility of using immune-related genes and cholesterogenic-related genes to construct a prostate cancer risk model combining immunity and cholesterol metabolism.

Consistent cluster analysis revealed that cholesterogenic-related genes could divide tumor samples into two subtypes. These results prompted us to further explore the correlation between cholesterogenic-related genes and prostate cancer prognosis. The KEGG and GO analyses showed that the cholesterogenic-related DEmRNAs were enriched in smooth muscle contraction. Patients with benign prostatic hyperplasia usually show dystonia of the smooth muscle of the prostate. Patients’ symptom scores can be improved by administering drugs that relax smooth muscle (Wang et al., 2016). Studies have shown that chronic prostatitis may be related to the development and progression of prostate cancer (De Nunzio et al., 2011). These cholesterogenic-related genes were also involved in the cGMP-PKG signaling pathway, which controls intracellular processes such as vasodilation and cell differentiation. GMP-dependent PKG2 inhibits the proliferation of cancer cells. It is reported that activation of the GMP-PKG pathway plays a role in prostate cancer (Wang et al., 2020).

Immune-related genes were involved in smooth muscle contraction and the cGMP-PKG signaling pathway. In the lipopolysaccharide-induced inflammatory microenvironment, knockdown of STEAP4 could suppress prostate cancer cell proliferation through the activation of the cGMP-PKG pathway (Li et al., 2021). A recent study also showed that low-dose metformin inhibits castration-resistant prostate cancer through modulating PDE6D-induced changes in purine metabolism and activating the cGMP-PKG pathway (Cai et al., 2025).

The regulation of metal ion transport was an enrichment function of the immune-related genes. It has been reported that the prostate is normally rich in zinc and that zinc reduction is a marker of prostate cancer development (To et al., 2020). The immune-related genes were also involved in focal adhesion, and focal adhesion kinase was involved in the occurrence of the cancer. Focal adhesion kinase is positively correlated with tumor stage and Gleason score in prostate cancer patients (Atılgan et al., 2020). The activation of the focal adhesion signaling pathway by MYO6 contributes to tumor progression in castration-resistant prostate cancer (Zheng et al., 2024). Overall, it can be speculated that the immune-related genes may contribute to prostate cancer via modulating these pathways.

Eleven genes (C2orf88, TRPM4, SAPCD2, RHPN1, RAC3, APOF, PTGS2, TSPAN1, KLK4, ENTPD5, and C1orf64) were selected by LASSO analysis to construct a risk model. It has been reported that C2orf88 may be a potential driver gene of prostate cancer (Peng et al., 2021), and the methylation of C2orf88 may be associated with prostate cancer (Babalyan et al., 2018). As a non-selective monovalent cation channel, TRPM4 expression can mediate the invasion and migration of cancer cells (Gao and Liao, 2019). TRPM4 was expressed in benign and malignant prostate tissues. Higher levels of TRPM4 were associated with higher rates of recurrence after prostatectomy (Berg et al., 2016). Silencing SAPCD2 could inhibit the proliferation, migration, and invasion of prostate cancer cells (Sun et al., 2021). RAC3 enhances the transcriptional activity of many steroid receptors. Moreover, endogenous RAC3 can interact with androgen receptors to increase their activity (Gnanapragasam et al., 2001).

The role of PTGS2 in various cancers has been widely reported. Further studies revealed that miR-124-3p inhibited the AKT/NF-κB pathway by targeting PTGS2 to play an antitumor effect in prostate cancer (Zhang, 2021). Some studies have reported that TSPAN1 is controlled by androgen, and higher TSPAN1 expression is associated with a lower Gleason score (Stinnesbeck et al., 2021). Munkley et al. also found that TSPAN1 expression was low in metastatic tumors (Munkley et al., 2017). KLK4 was found to remodel the prostate tumor microenvironment (Gao et al., 2007). ENTPD5 is typically expressed only in prostate tumors compared to normal samples (de Campos et al., 2021). Overexpression of RHPN1 is found to be linked to poor prognosis in prostate adenocarcinoma (Hou et al., 2021a). APOF plays a key role in lipid metabolism, and this process has been associated with the risk of multiple cancers, including prostate cancer (Marrone et al., 2023; Shi et al., 2023). C1orf64 (also named SRARP) is a tumor suppressor that can be used to predict the clinical outcomes of malignant tumors, potentially including prostate cancer (Marrone et al., 2023).

In this study, a prognostic risk model constructed by these immune-cholesterogenic-related genes demonstrated a good ability for predicting prostate cancer prognosis. Determining the expression levels of these genes may help identify high-risk patients who are more likely to experience poor outcomes, enabling personalized treatment plans. Additionally, the model could be integrated into current diagnostic protocols by combining gene expression data with other clinical and molecular biomarkers, enhancing the accuracy. While this prognostic risk model demonstrated promising predictive performance, its practical application in clinical settings can be further realized by integrating it into existing diagnostic protocols and treatment strategies. This would ultimately contribute to the advancement of precision medicine for patients with prostate cancer.

We constructed a nomogram combined with the Gleason score and the risk score. The nomogram showed great prognostic potential. We also found that the proportion of T3 was highest in the high-risk group, while the proportion of T2 was highest in the Low_risk group. The K–M curve of the risk model indicated that patients in the High_risk group had a worse prognosis. Patients in the High_risk group were older, had higher Gleason scores, and had more patients in the N1 stage. This is consistent with previous research findings (Hall et al., 2005).

Increasing evidence has shown that various immune cells are implicated in the progression of prostate cancer (Novysedlak et al., 2025). Immune cell infiltration into prostate tissue has been associated with prostate cancer progression and lipid metabolism disturbances (Siltari et al., 2022). The lipid-mediated crosstalk between cancer cells and immune cells in the tumor microenvironment can affect immune cell functions and plays an essential role in cancer progression and immune evasion (Zeng et al., 2022). Cholesterol metabolism may influence the tumor microenvironment by affecting immune cell function and infiltration. In prostate cancer, such metabolic shifts could affect the immune microenvironment by promoting immune suppression or resistance to immunotherapies.

We analyzed the relationship between immune cell infiltration and the risk model. Immune infiltration analysis indicated that M2 macrophages and Tregs showed a significantly higher fraction in the High_risk group. M2 macrophages are thought to influence disease outcomes by stimulating angiogenesis and immunosuppression. An increased number of Tregs in men with prostate cancer always accompanies a poor prognosis and reduced survival (Erlandsson et al., 2019). Therefore, we conclude that the infiltration of these immune cells may affect prostate cancer progression and patient outcomes. Notably, a promising strategy for therapeutic intervention involves tumor cell membrane-based vaccines, which utilize tumor-derived membrane vesicles to stimulate antitumor immunity (Yang et al., 2024). Given that lipid molecules, including cholesterol, affect cell membrane properties, it is plausible that metabolic modulation of these membranes could optimize the presentation of tumor antigens and increase the efficacy of tumor vaccines. By integrating metabolic biomarkers with immune-targeting therapies, a new avenue for precision immunotherapy could be realized, where treatment is fine-tuned to both the metabolic state of the tumor and the immune landscape of the patient.

This study identified seven key immune-cholesterogenic-related genes and successfully developed a prognostic model integrating cholesterol metabolism and immune-related gene signatures for prostate cancer, offering valuable insights into the potential of metabolic-immune crosstalk in shaping tumor progression and patient outcomes. However, there were some limitations. First, sequencing data and clinical data were obtained from public databases. The results were not validated by clinical cohorts. Second, this was a retrospective study, which may introduce potential biases, such as selection bias and unmeasured confounders. Further prospective studies are required to validate these findings. Third, this study did not account for potential confounders that could influence cholesterol levels and immune responses, such as patient comorbidities or lifestyle factors such as diet and smoking. Future studies that incorporate these confounders are needed to better assess the relationship between lipid metabolism and immune response in prostate cancer. Finally, while this study identified prognostic genes and pathways, their functional roles in prostate cancer were not experimentally validated. Functional studies, such as *in vitro* or *in vivo* experiments, are necessary to further support their biological conclusions.

In conclusion, we constructed a risk model based on cholesterogenic-related and immune-related genes to predict the prognosis of prostate cancer. The model and the nomogram had great prognostic performance. These immune-cholesterol-related genes and the model may serve as valuable prognostic biomarkers, guiding personalized treatment strategies for prostate cancer patients.

## Data Availability

The TCGA-Prostate Cancer data used for analysis in the study are deposited in the TCGA repository. The data used for validation are deposited in the Gene Expression Omnibus (GEO) repository, accession number GSE70769.

## Supplementary material

SUPPLEMENTARY FIGURE 1 The flow chart.

SUPPLEMENTARY FIGURE 2 Proportions of clinical factors in different groups. **(A)** Proportions of different pathologic_T in different groups. **(B)** Proportions of different ages in different groups. **(C)** Proportions of different Gleason scores in different groups. **(D)** Proportions of different pathologic_N in different groups.

SUPPLEMENTARY FIGURE 3 Analysis of the immunocyte infiltration degrees in two groups regarding immunocyte subunits. Green represents the Low_risk group, and red represents the High_risk group.

SUPPLEMENTARY FIGURE 4 The mutation frequency of genes in patients with PDAC. **(A)** The mutation frequency of genes in patients in the High_risk group. **(B)** The mutation frequency of genes in patients in the Low_risk group.
