# Supplementary figures and images for: Developing prognostic models for cholesterol-related genes linked with immune infiltration in prostate cancer
Source: Front Genet. 2025 Sep 26;16:1604113. doi: 10.3389/fgene.2025.1604113 (PMC12510679; doi:10.3389/fgene.2025.1604113)

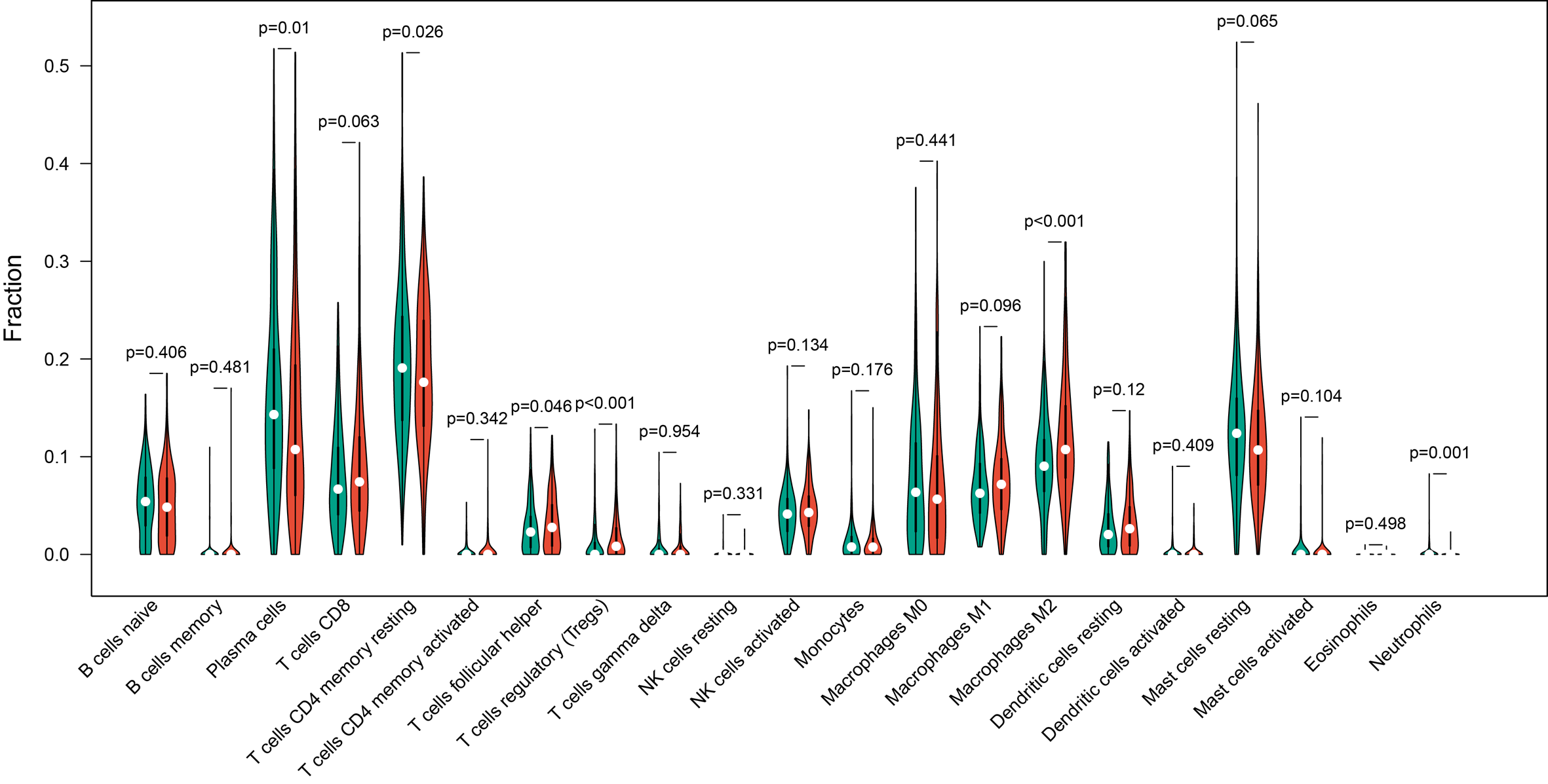

Supplement: Supplementary file 1 [file Image3.tif]

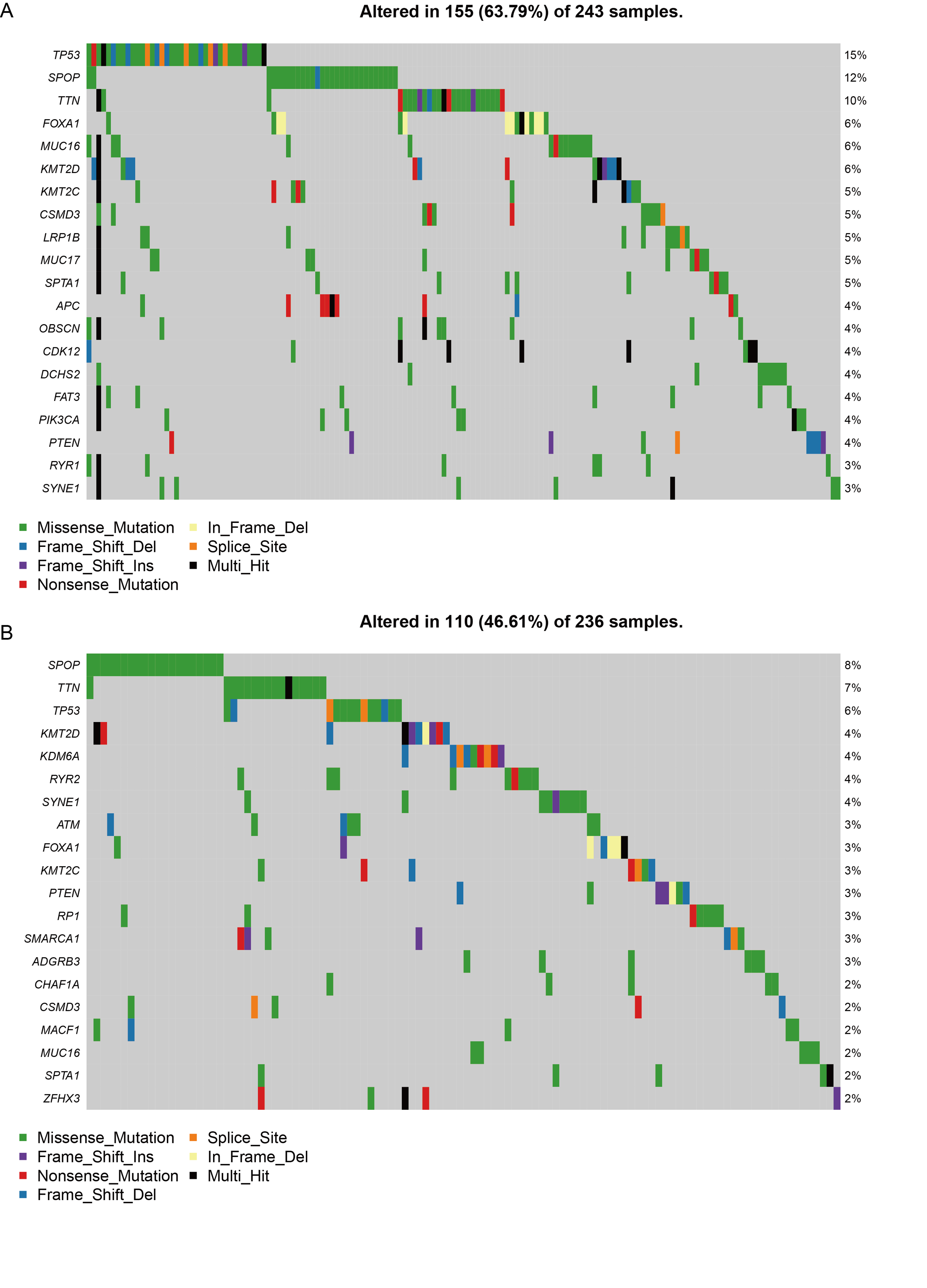

Supplement: Supplementary file 2 [file Image4.tif]

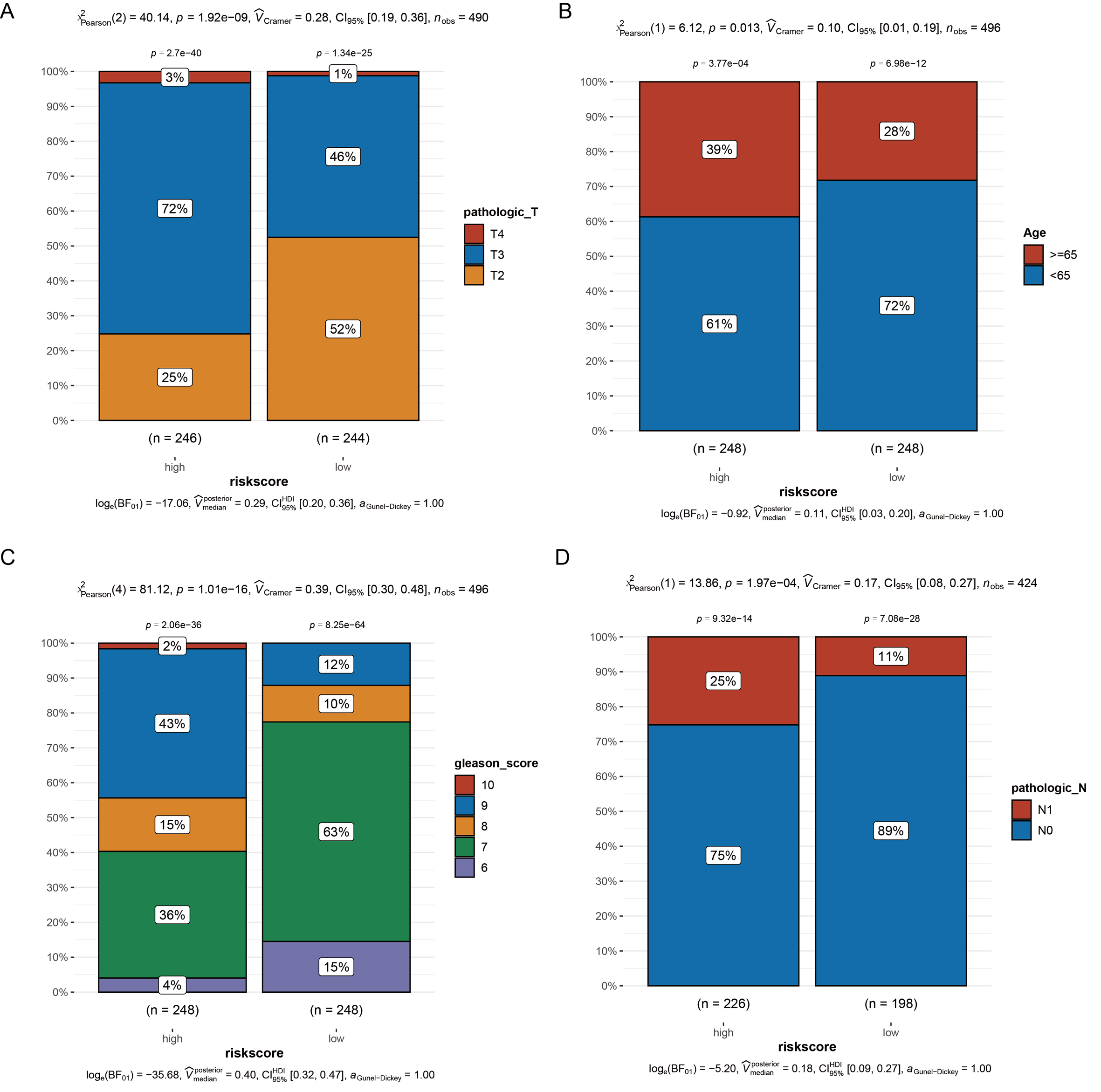

Supplement: Supplementary file 3 [file Image2.tif]

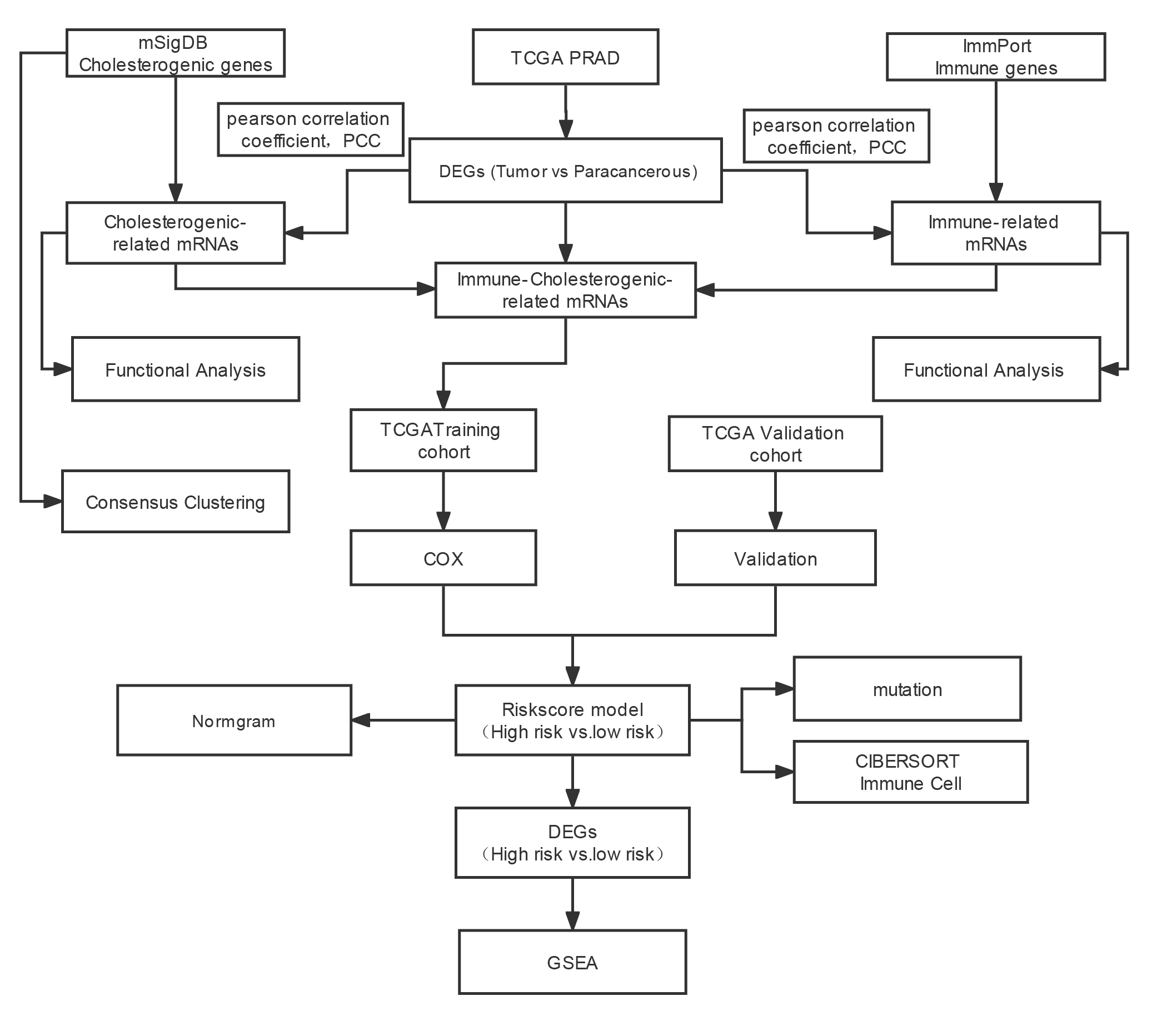

Supplement: Supplementary file 4 [file Image1.tif]
